# Supplementary material for: Challenging the Conventional Interpretation of HCMV Seronegativity
Source: Microorganisms. 2021 Nov 18;9(11):2382. doi: 10.3390/microorganisms9112382 (PMC8626044; doi:10.3390/microorganisms9112382)
Supplement: Supplementary file 1 [file microorganisms-09-02382-s001.zip › microorganisms-1449382-supplementary.pdf]

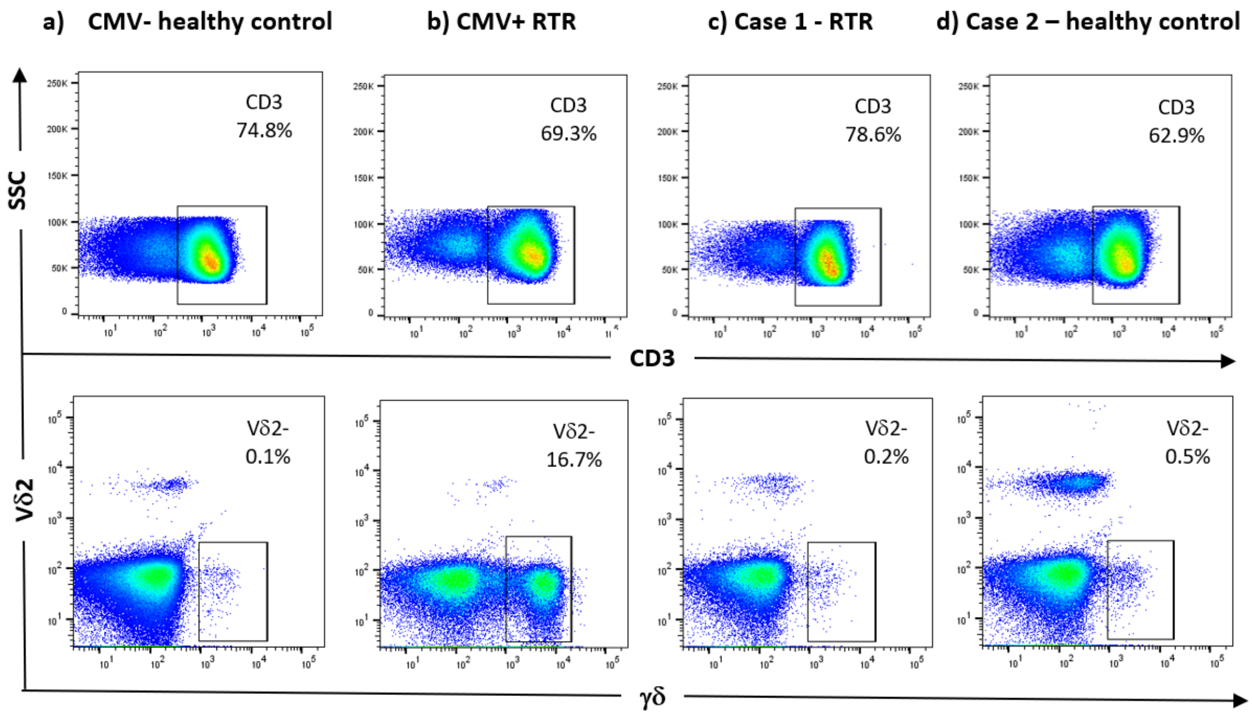

Supplementary Figure S1. Vδ2 expression on T-cells. Representative flow cytometry plots for a CMV seronegative healthy control (a) and a CMV seropositive renal transplant recipient (RTR) (b), Case 1 (c) and Case 2 (d). Gating strategy: singlets were first defined by forward scatter area (FSC-A) and forward scatter height, lymphocytes were then gated based on side scatter area and FSC-A, and dead cells were excluded based on uptake of Fixable Viability Stain. Lack of Vδ2 expression (bottom panel) was assessed in T-cells identified as $CD3^+$ (top panel). The following antibodies were used - anti-CD3 APC (clone UCHT1, anti-TCRγ/δ-1 PECy7 (clone 11F2, San Jose, CA), and anti-Vδ2 TCR FITC (clone B6) from BD Bioscience. Data were acquired on a BD LSR II Fortessa instrument (BD Bioscience, Ashland, OR).
